# Effect of the Non-Immunosuppressive MPT Pore Inhibitor Alisporivir on the Functioning of Heart Mitochondria in Dystrophin-Deficient *mdx* Mice

**DOI:** 10.3390/biomedicines9091232

**Published:** 2021-09-16

**Authors:** Mikhail V. Dubinin, Vlada S. Starinets, Eugeny Yu. Talanov, Irina B. Mikheeva, Natalia V. Belosludtseva, Dmitriy A. Serov, Kirill S. Tenkov, Evgeniya V. Belosludtseva, Konstantin N. Belosludtsev

**Affiliations:** 1 Department of Biochemistry, Cell Biology and Microbiology, Mari State University, pl. Lenina 1, 424001 Yoshkar-Ola, Russia; vlastar@list.ru (V.S.S.); kirill.tenkove@gmail.com (K.S.T.); shaila2009@mail.ru (E.V.B.); bekonik@gmail.com (K.N.B.); 2 Laboratory of Mitochondrial Transport, Institute of Theoretical and Experimental Biophysics, Russian Academy of Sciences, Institutskaya 3, 142290 Pushchino, Russia; evg-talanov@yandex.ru (E.Y.T.); mikheirina@yandex.ru (I.B.M.); nata.imagination@gmail.com (N.V.B.); 3 Prokhorov General Physics Institute of the Russian Academy of Sciences, 38 Vavilov St., 119991 Moscow, Russia; dmitriy_serov_91@mail.ru

**Keywords:** Duchenne muscular dystrophy, heart, alisporivir, mitochondria, mitochondrial permeability transition

## Abstract

Supporting mitochondrial function is one of the therapeutic strategies that improve the functioning of skeletal muscle in Duchenne muscular dystrophy (DMD). In this work, we studied the effect of a non-immunosuppressive inhibitor of mitochondrial permeability transition pore (MPTP) alisporivir (5 mg/kg/day), reducing the intensity of the necrotic process and inflammation in skeletal muscles on the cardiac phenotype of dystrophin-deficient *mdx* mice. We found that the heart mitochondria of *mdx* mice show an increase in the intensity of oxidative phosphorylation and an increase in the resistance of organelles to the MPT pore opening. Alisporivir had no significant effect on the hyperfunctionalization of the heart mitochondria of *mdx* mice, and the state of the heart mitochondria of wild-type animals did not affect the dynamics of organelles but significantly suppressed mitochondrial biogenesis and reduced the amount of mtDNA in the heart muscle. Moreover, alisporivir suppressed mitochondrial biogenesis in the heart of wild-type mice. Alisporivir treatment resulted in a decrease in heart weight in *mdx* mice, which was associated with a significant modification of the transmission of excitation in the heart. The latter was also noted in the case of WT mice treated with alisporivir. The paper discusses the prospects for using alisporivir to correct the function of heart mitochondria in DMD.

## 1. Introduction

Duchenne muscular dystrophy (DMD) is a rare (1:3500 male births) inherited X-linked recessive disorder [1,2]. The cause of the disease is mutations in the gene encoding a set of dystrophin proteins, the largest of which reaches 427 kDa and is involved in the formation of the dystrophin–glycoprotein complex in cardiac and skeletal muscle cells. This complex provides the connection of the cytoskeleton of muscle cells with the extracellular matrix, maintaining the structural integrity of the tissue and its functional activity, and its absence is accompanied by progressive destabilization of the muscle fiber [3]. The main and primary clinical manifestations of DMD are associated with skeletal muscle weakness; however, the development of severe cardiac dysfunction by the third decade of life in patients is known [4].

It is believed that the rapid progression of destructive processes in muscle tissue is associated with significant dysfunction of intracellular organelles—the sarcoplasmic reticulum and mitochondria [3]. Indeed, it is known that in Duchenne dystrophy, skeletal muscle mitochondria show a decrease in the intensity of oxidative phosphorylation, ROS overproduction, as well as impairment of the ability to accumulate a large amount of Ca^2+^ in the matrix due to a decrease in the resistance of organelles to the induction of mitochondrial permeability transition (MPT) pore and rearrangements of calcium-transporting systems [5,6,7]. The latter is especially important in the case of DMD, characterized by elevated levels of intracellular Ca^2+^ [8] contributing to calpain activation followed by degradation of muscle proteins. Therefore, it is not surprising that one of the promising strategies proposed for the correction of muscle dysfunction in DMD is mitochondria-targeted therapy associated with maintaining the functional activity of organelles. In particular, it was previously shown that the prevention of MPT pore opening in the inner membrane of organelles, using pharmacological inhibitor cyclosporin A (CsA) and its analog alisporivir (also known as Debio 025), is capable of blocking the activity of cyclophilin D matrix enzyme mediating MPTP opening significantly, which improves pathology in preclinical animal models, reduces the level of fibrosis in skeletal muscles and the intensity of necrosis and inflammation, and normalizes the functional activity of mitochondria [9,10,11,12]. In this regard, alisporivir seems to be the most promising drug since, unlike CsA, it does not have immunosuppressive properties and does not inhibit calcineurin signaling [13], which is known to secondarily reduce myotube differentiation and muscle regeneration [14].

Along with this, the effect of MPT pore inhibition and alisporivir therapy on cardiac muscle dysfunction in DMD is unknown. Earlier, other groups and we found significant changes in the nature of mitochondrial dysregulation in the cardiac muscle of *mdx* animals, which contrasted with a decrease in the functional activity of organelles in skeletal muscles already at the early stages of the development of pathology. On the contrary, in this case, we noted an increase in the functional activity of heart mitochondria accompanied by a high intensity of oxidative phosphorylation and the ability to transport and retain calcium ions in the matrix of organelles [15,16,17]. We hypothesized that such a picture might contribute to the adaptation of the heart to the development of muscular dystrophy and delay cardiac pathology.

In this work, we first evaluated the effect of alisporivir treatment on the functional activity of heart mitochondria of dystrophin-deficient C57BL/10ScSn-Dmdmdx line (*mdx* mice) and the wild-type C57BL/10 line (wild-type mice), as well on the dynamics and biogenesis of organelles. The results obtained indicate that alisporivir maintains an increased functional activity of the cardiac mitochondria of *mdx* mice, which can have a significant effect on the state and function of the heart muscle.

## 2. Materials and Methods

### 2.1. Animals

The mice used were C57BL10 mice (wild-type, WT) and dystrophin-deficient mdx (C57BL/10ScSn-mdx). All the animals were purchased from the Animal Breeding Facility, Branch of the Shemyakin and Ovchinnikov Institute of Bioorganic Chemistry, Russian Academy of Sciences, Russia (IBCh RAS Unique Research Device “Bio-model”). Upon arrival, mice were singly housed and given a minimum of 72 h to acclimatize before experiments were performed. All animals were provided access to standard chow and water ad libitum. The *mdx* and wild-type mice were divided into four treatment groups (*n* = 10 per group): (1) vehicle-treated wild-type mice (WT), (2) WT + alisporivir (WT + Ali), (3) *mdx* mice, and (4) *mdx* mice treated with alisporivir (*mdx* + Ali). We treated *mdx* and wild-type mice beginning at 8 weeks of age. Alisporivir (1 mg/mL, Medchemexpress, Monmouth Junction, NJ, USA, cat. no. HY-12559) was dissolved in a mixture of DMSO, ethanol, and sterile saline (12.5:25:62.5 *v*/*v*%) and administered in doses of 150–200 μL (5 mg/kg body weight) per mouse intraperitoneally every day for up to 4 weeks. Sham-injected controls received solvent alone. At the end of the treatment period, all mice were sacrificed, and body/heart weights were recorded. Blood was collected at the end of all studies for analysis of creatine kinase, lactate dehydrogenase (LDH), and aspartate aminotransferase (AST) levels using the appropriate reagent kits (Vector-Best, Novosibirsk, Russia). Mitochondrial isolation was performed in fresh samples of the heart muscle. The rest of the tissue was stored at −80 °C until analyzed.

### 2.2. ECG

The combined anesthesia was used for the immobilization of animals [18]. The mice were preconditioned for about 5 min with gas mixture N_2_O:O_2_ (77%:23%) (Akela-N, Russia). After precondition, the mice were intraperitoneally injected with 50 μg/kg “Zoletil” (Zoletil 100, Virbac Sante Animale, France). The N_2_O:O_2_ mixture was fed in the face mask during the whole time of the experiment. A mouse was held at rest until it lost a righting reflex and then was placed in the experimental chamber. Measurements were started only after the negative tail pinch reflex test to guarantee surgical anesthesia depth [19,20].

Electrocardiograms (EGCs) were recorded in the II standard lead for 10 min with the SparkFun Single Lead Heart Rate Monitor (SparkFun Electronics, Niwot, CO, USA). ECG signals were processed with Clampfit 11.2.0.59 software (Molecular Devices LLC, San Jose, CA, USA). ECG signals were filtered with Highpass Bessel filter (cutoff frequency 0.14 Hz) Lowpass Gaussian filter (cutoff frequency 45 Hz). QRS complex duration and R/S ratios were evaluated for each ECG record [21].

### 2.3. Electron Microscopy

For the electron microscopy examination, pieces of the left ventricles of two hearts of each experimental group of animals were taken and fixed in a 2.5% glutaraldehyde solution in 0.1 M phosphate-buffered saline (PBS, pH 7.4) for 2 h. After washing with the buffer, the tissue was fixed for 2 h with a 1% solution of osmium acid in PBS and dehydrated by increasing concentrations of alcohols. The resulting samples were encapsulated in Epon 812 resin. Ultrathin sections (70–75 nm) were prepared on a Leica EM UC6 microtome (Leica Microsystems, Wetzlar, Germany) and stained with uranyl acetate and lead citrate. The preparations were viewed and photographed using a JEM-100B electron microscope (JEOL, Tokyo, Japan). Ultrastructural analysis was performed using negative images digitized with an Epson V700 scanner (Seiko Epson Corporation, Nagano, Japan).

### 2.4. Mitochondria Isolation and Determination of Respiration and Oxidative Phosphorylation

Mitochondria were isolated from heart muscles using a convenient technique of differential centrifugation, as previously described [16]. The isolation medium contained 67 mM Sucrose, 50 mM KCl, 10 mM EDTA, 0.2% BSA, and 50 mM Tris/HCl buffer (pH 7.4). The resulting suspension of mitochondria was resuspended in 250 mM Sucrose and 10 mM Tris/HCl buffer (pH 7.4) and contained 20–30 mg of mitochondrial protein/mL, as determined by the Lowry method. The rate of oxygen consumption was measured polarographically with a Clark-type gold electrode and Oxygraph-2k (Oroboros Instruments, Innsbruck, Austria) at 25 °C under continuous stirring. The reaction medium contained 120 mM KCl, 5 mM NaH_2_PO_4_, 2.5 mM potassium malate, 2.5 mM potassium glutamate, 10 mM Hepes/KOH, pH 7.4. Other reagents: 0.2 mM ADP and 50 μM 2,4-dinitrophenol (DNP). The assessment of the functional parameters of mitochondrial respiration was carried out by the generally accepted method [22]. The concentration of mitochondrial protein was 0.25 mg/mL.

### 2.5. Determination of Ca^2+^ Retention by Mitochondria, MPT Pore Opening Assay

The transport of Ca^2+^ across the inner membrane of isolated organelles was monitored with an arsenazo III (2,2′-(1,8-Dihydroxy-3,6-disulfonaphthylene-2,7-bisazo) bisbenzenearsonic acid, 2,7-Bis(2-arsonophenylazo)chromotropic acid) indicator at 675–685 nm using a plate reader Tecan Spark 10M (Tecan Group Ltd., Männedorf, Switzerland) at 25 °C under constant stirring, as previously described [6,7,16]. To determine the ability of mitochondria (0.25 mg of mitochondrial protein/mL) to retain Ca^2+^, 10 μM CaCl_2_ was successively added into the reaction medium 210 mM mannitol, 70 mM sucrose, 1 mM KH_2_PO_4_, 2.5 mM potassium malate, 2.5 mM potassium glutamate, 50 μM arsenazo III, 10 μM EGTA, and 10 mM HEPES-KOH (pH 7.4). After several additions, external [Ca^2+^] increased, indicating a massive release of the ion from the organelles due to the opening of the MPT pore. The amount of Ca^2+^ released upon permeability transition (defined as Ca^2+^ retention capacity (CRC) was used as a measure of the MPT pore opening probability.

### 2.6. Lipid Peroxidation

Lipid peroxidation in a suspension of isolated mitochondria was estimated spectrophotometrically by measuring the levels of thiobarbituric acid-reactive substances (TBARS). The TBARS assay quantifies the levels of malondialdehyde and other minor aldehyde species through their reaction with thiobarbituric acid [23].

### 2.7. RNA Extraction, Reverse Transcription, and Quantitative Real-Time PCR

Total RNA was isolated from 100 mg of deep-frozen tissue samples using an ExtractRNA kit (#BC032, Eurogen, Moscow, Russia) in accordance with the protocol of the manufacturer. The real-time PCR was performed on a DTLite5 amplifier (DNA-Technology LLC, Moscow, Russia) using the qPCRmix-HS SYBR reaction mixture (Eurogen, Moscow, Russia). The selection and analysis of gene-specific primers were performed using Primer-BLAST [24] (the oligonucleotide sequences are presented in Table 1). The relative level of expression of each gene was normalized to the level of *Rplp2* mRNA, and a comparative C_T_ method was used to quantify the results [25].

### 2.8. Quantification of Mitochondrial DNA

Total DNA (nuclear and mtDNA) was extracted from 10 mg of heart tissue using DNA-Extran 2 kit (Sintol, Moscow, Russia) in accordance with the protocol of the manufacture; 1 ng of the total DNA was taken for the reaction. Evaluation of mtDNA content in cardiac tissue was performed by PCR as described [26] and expressed as mtDNA/nuclear DNA ratio. For our assay, we selected the ND4 gene of the mouse mitochondrial genome and GAPDH, which is a nuclear-encoded gene. A comparison of ND4 DNA expression relative to GAPDH DNA expression will give a measure of mtDNA copy number to nDNA copy number ratio. Primers for mtDNA and nDNA are presented in Table 1. The real-time PCR was performed with a DTLite5 amplifier (DNA-Technology LLC, Moscow, Russia) using the qPCRmix-HS SYBR reaction mixture (Evrogen, Moscow, Russia), which contained a commonly used fluorescent DNA binding dye SYBR Green II.

### 2.9. Electrophoresis and Immunoblotting of Mitochondrial OXPHOS Proteins

Total protein extracts were prepared from 10 mg of the frozen heart muscle. To maintain extract integrity and function, Complete Protease Inhibitor Cocktail (P8340, Sigma-Aldrich, USA), Phosphatase Inhibitor Cocktail 3 (P0044 Sigma-Aldrich, St. Louis, MO, USA), PMSF (1 mM), Na_3_VO_4_ (1 mM), EGTA (1 mM), EDTA (1 mM) were used. Proteins were isolated using a RIPA buffer (20-188, Merck Millipore Ltd., Billerica, MA, USA). Quick Start Bradford Protein Assay (Bio-Rad Laboratories, Hercules, CA, USA) was used to quantify protein content. The samples were diluted in Laemmli buffer, run on 12.5% SDS-PAGE (10 µg/lane), and transferred to a 0.45 µm nitrocellulose membrane (Cytiva, Marlborough, MA, USA). After overnight blocking, the membrane was incubated with the appropriate primary antibody. The total OXPHOS Rodent WB Antibody Cocktail (ab110413) and Anti-alpha Tubulin antibody (ab4074) were from Abcam. The immunoreactivity was detected using the appropriate secondary antibody conjugated to horseradish peroxidase (7074, Cell Signaling Technology Inc., (Danvers, MA, USA). Peroxidase activity was detected with ECL chemiluminescence reagents (Pierce, Rockford, IL, USA). The relative levels of the detected proteins were visualized using an LI-COR system (LI-COR, Lincoln, NE, USA) and normalized to the alpha tubulin loading control. Optical density measurements were performed by LI-COR Image Studio software.

### 2.10. Statistical Analysis

The data were analyzed using the GraphPad Prism 6.0 software (GraphPad Software Inc., La Jolla, CA, USA) and were presented as mean ± SEM of 4–10 biological replicates (excluding electron microscopy data). The results of the transmission electron microscopy analysis were presented as representative images from two biological replicates. The statistical significance of the differences between the experimental groups was evaluated using a one-way analysis of variance (ANOVA) followed by the Tukey multiple comparison post hoc test.

## 3. Results

### 3.1. Effect of Alisporivir on the Intensity of the Inflammatory Process in mdx Mice

At the first stage of the work, we evaluated the effect of alisporivir treatment on the inflammatory status of *mdx* mice and measured the serum level of enzymes reflecting the intensity of the inflammatory process in experimental groups of animals. Indeed, it is known that the destruction of the cell membranes of *mdx* mice and the development of the necrotic process leads to the release of intracellular enzymes—creatine kinase, lactate dehydrogenase (LDH), and aspartate aminotransferase (AST) into the serum, and a significant increase in their level [10,11]. Twelve-week *mdx* mice also show this pattern (Table 2). In this case, alisporivir administration does not affect the level of creatine kinase and LDH but significantly reduces the level of AST. This is consistent with previously obtained data [10,11] and may indicate a partial decrease in the intensity of the inflammatory process in the muscle tissue of alisporivir-treated dystrophin-deficient animals.

### 3.2. Effect of Alisporivir on the Ultrastructure of Heart Mitochondria

**Figure 1:**
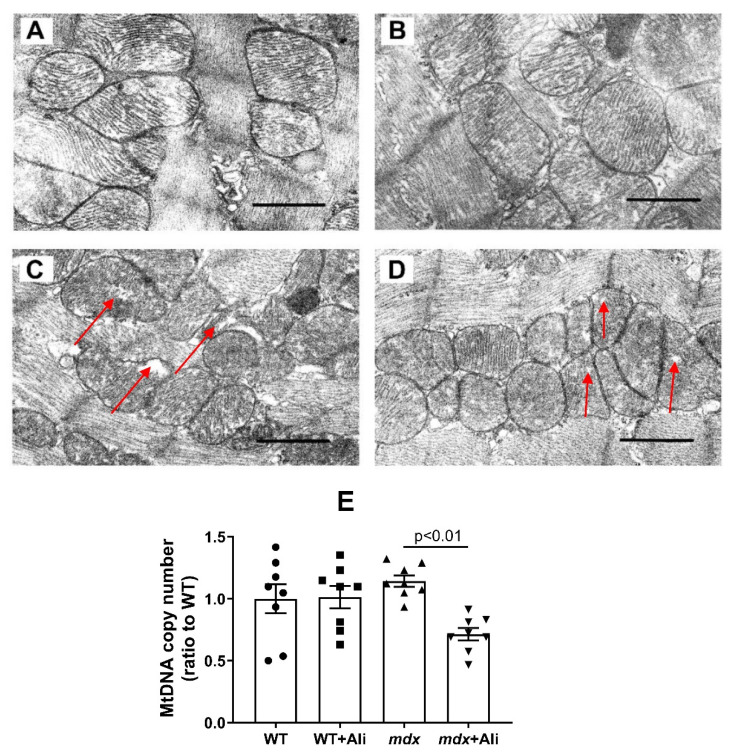
Typical electron micrographs of left ventricular mitochondria in the experimental groups: WT (**A**), WT + Ali (**B**), *mdx* (**C**), and *mdx* + Ali (**D**). Samples from two hearts were analyzed in each experimental group. The bar is equal to 1 μm. Red arrows indicate individual abnormal mitochondria. MtDNA copy numbers (**E**). Data are presented as means ± SEM (*n* = 6).

Figure 1 and Figure S1 show representative photomicrographs of cardiac myocyte mitochondria from four experimental groups. In the heart muscle, the mitochondria of the control group (WT) are represented by bean-shaped and rounded structures with numerous cristae surrounded by an intact outer membrane (Figure 1A and Figure S1a,b). The ultrastructural organization of mitochondria in the WT + Ali group does not differ from the control one (Figure 1B and Figure S1c,d). Mitochondria from *mdx* mice are spherical structures with an irregular organization of mitochondrial cristae (Figure 1C and Figure S1e,f). There are some mitochondria with vacuoles and abnormalities in the outer membrane. The mitochondrial morphology in the *mdx* + Ali group is similar to the WT and WT + Ali groups (Figure 1D and Figure S1g,h). However, a slight vacuolization of mitochondria remains.

Along with some disruption of the ultrastructure of the heart mitochondria in *mdx* mice, the number of organelles, estimated by mtDNA copy, does not change compared to wild-type animals (Figure 1E). We have previously obtained similar results [16]. However, the level of mtDNA in the heart significantly decreases in the *mdx* + Ali group.

### 3.3. Impact of Alisporivir on the Functioning of Heart Mitochondria

**Figure 2:**
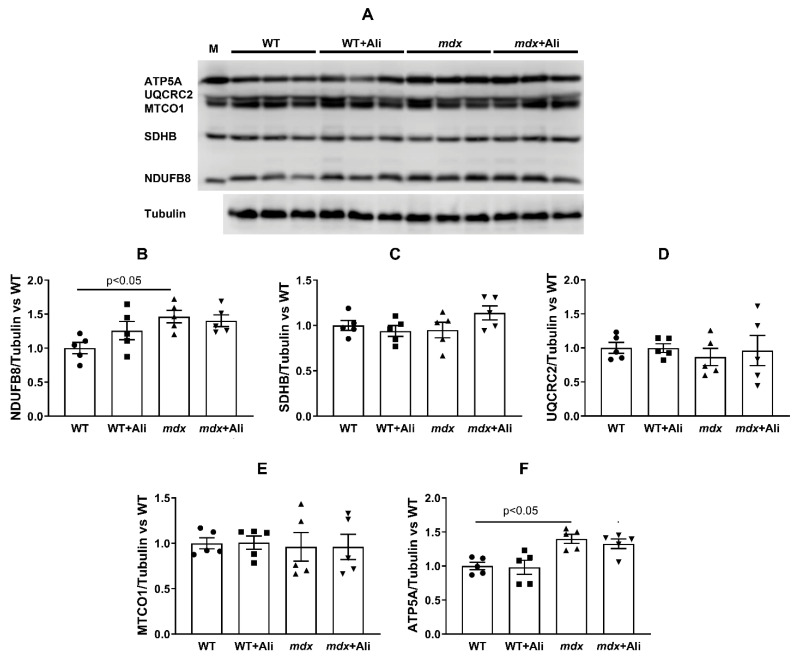
Levels of the proteins of mitochondrial respiratory chain complexes. Data of Western blot analysis (**A**). The letter “M” indicates a positive control (rat heart tissue lysate; mitochondrial extract). Relative contents of Complex I/tubulin ratio (**B**), Complex II/tubulin ratio (**C**), Complex III/tubulin ratio (**D**), Complex IV/tubulin ratio (**E**), and Complex V/tubulin ratio (ATP synthase, (**F**)). The data are presented as means ± SEM (*n* = 5).

In the next part of the work, we evaluated the effect of alisporivir administration on the parameters of glutamate/malate-fueled respiration and oxidative phosphorylation of cardiac mitochondria in mice from four experimental groups. We previously found that the cardiac mitochondria of 4-week *mdx* mice show an increase in the intensity of respiration and oxidative phosphorylation [16]. Table 3 demonstrates that 12-week *mdx* mice maintain this trend. Indeed, one can see that the heart mitochondria of dystrophin-deficient animals show a 1.6-fold increase in the rate of ADP-stimulated respiration (state 3) and a 1.7-fold increase in the maximum respiration rate in the presence of the protonophore uncoupler DNP (state 3U_DNP_) compared with wild-type animals. This is also accompanied by a 1.3-fold increase in the respiratory control ratio. In this case, alisporivir has no significant effect on the functional activity of the heart mitochondria of *mdx* and WT mice. To determine the cause of the change in the rate of mitochondrial respiration, we estimated the content of proteins that build up the complexes of the respiratory chain by Western blotting in mitochondria of four experimental groups. Figure 2 demonstrates that the heart mitochondria of *mdx* mice show an increase in the level of complex I as well as complex V (α-subunit of ATP synthase, ATP5A) compared to wild-type mice. In this case, administration of alisporivir to *mdx* mice (*mdx* + Ali group) does not lead to a change in the level of this protein compared to the *mdx* group. We also did not observe the effect of alisporivir on the level of OXPHOS proteins in WT mice. The levels of other complexes of the respiratory chain of organelles do not change.

**Figure 3:**
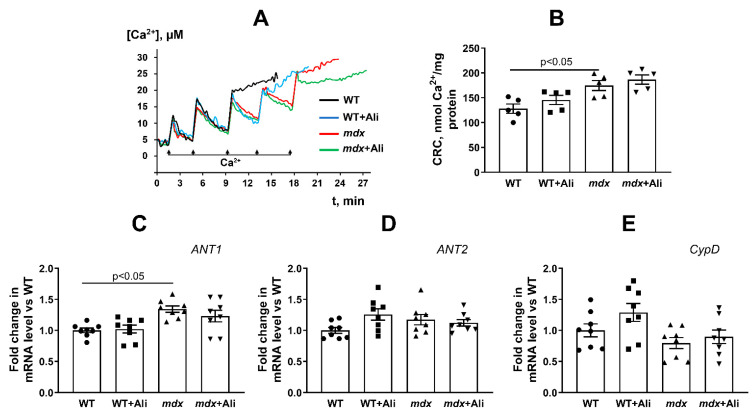
The opening of Ca^2+^-dependent MPT pore in the heart mitochondria of studied groups of mice. Uptake of sequential Ca^2+^ additions by the heart mitochondria (**A**). Pulse additions of 10 μM Ca^2+^ are indicated by arrows. The figure shows traces of a typical experiment conducted at the same time on the same mitochondrial preparation. Similar results were obtained in 5 independent experiments. Ca^2+^ retention capacity (CRC) of heart mitochondria of studied groups of mice (**B**). The data are presented as means ± SEM (*n* = 5). Gene expression of MPT-related proteins measured by real-time PCR: *ANT1* (**C**), *ANT2* (**D**), *CypD* (**E**). The data are presented as means ± SEM (*n* = 8).

We also evaluated the resistance of the heart mitochondria of experimental groups of mice to the induction of a calcium-dependent MPT pore. One can see that the mitochondria of *mdx* mice show a 1.4-fold increase in Ca^2+^ retention capacity compared to the mitochondria of wild-type animals (Figure 3A,B). This is consistent with previously obtained data [16] and indicates an increase in the resistance of heart mitochondria of dystrophin-deficient mice to MPT pore opening. In this case, alisporivir administration does not affect the Ca^2+^ capacity of heart mitochondria in *mdx* mice, as well as in WT animals. We assessed the level of proteins—putative components of the MPT pore (ANT1, ANT2, and CypD) in the heart mitochondria of experimental groups of animals. Figure 3C–E shows that the expression of genes encoding ANT2 and cyclophilin D is not altered in the heart of *mdx* mice. At the same time, the heart muscles of *mdx* mice show a 1.3-fold increase in ANT1 expression compared to WT animals. Alisporivir administration does not significantly affect the expression of these genes in the heart muscles of experimental groups of animals.

**Figure 4:**
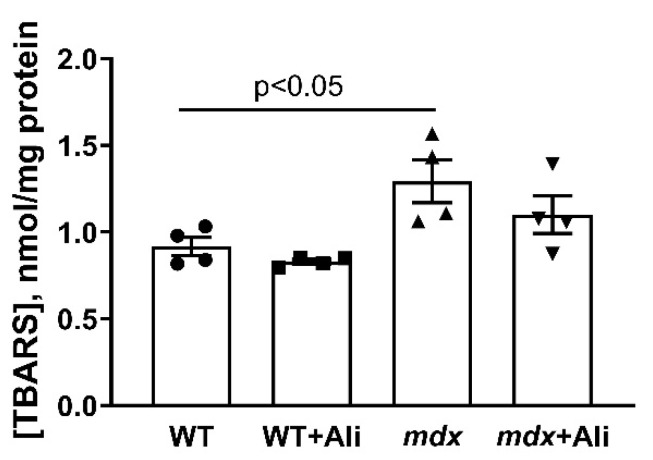
Effect of alisporivir on lipid peroxidation in mouse heart mitochondria. Lipid peroxidation was assessed by the level of TBARS in the heart mitochondria of experimental groups of animals. The data are presented as means ± SEM (*n* = 4).

We previously showed that the heart mitochondria of dystrophin-deficient mice are characterized by an increase in ROS production [16], which may contribute to oxidative damage to organelles and other structures of the muscle fiber. In this work, we also noted an increase in the intensity of oxidative stress in the heart mitochondria of *mdx* mice, as evidenced by an increase in the level of thiobarbituric reactive substances (malondialdehyde) compared to control animals (Figure 4). Alisporivir administration to animals of both groups has no effect on this parameter.

### 3.4. Effect of Alisporivir on the Expression of Proteins Responsible for Mitochondrial Biogenesis and Mitochondrial Dynamics

**Figure 5:**
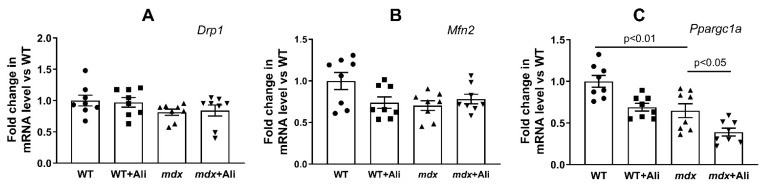
The relative mRNA levels of *Drp1* (**A**), *Mfn2* (**B**), *Ppargc1a* (**C**) in the heart of experimental animals. The data are presented as means ± SEM (*n* = 8).

Mitochondrial dysfunction in DMD is known to be associated with altered mitochondrial dynamics and biogenesis [27,28]. Here, we evaluated the expression of genes (*Drp1*, *Mfn2*, and *Ppargc1a*) encoding proteins responsible for mitochondrial fusion, fission, and mitochondrial biogenesis, respectively. One can see that the heart muscle of dystrophin-deficient mice is characterized by a decrease in the expression of the *Ppargc1a* gene in comparison with the wild-type mice (Figure 5). At the same time, the expression of the mitofusin 2 gene and the *Drp1* gene does not change significantly. In this case, dystrophin-deficient mice treated with alisporivir show a significant decrease in *Ppargc1a* expression compared to *mdx* mice. *Drp1* and mitofusin 2 levels do not change. This indicates the ability of alisporivir to suppress mitochondrial biogenesis in the heart of *mdx* mice.

### 3.5. Impact of Alisporivir on Some Parameters of the Heart Muscle

**Figure 6:**
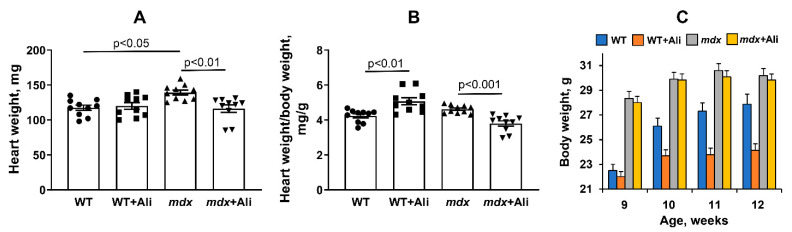
The effect of alisporivir administration on absolute (**A**) and relative heart weight (**B**), as well as on body weight gain (**C**) in experimental groups of mice. The data are presented as means ± SEM (*n* = 10).

Finally, we evaluated the effect of the alisporivir administration on the state and functioning of the heart of experimental groups of animals. It is known that DMD shows the development of heart muscle hypertrophy [29]. The *mdx* mice also reveal this picture. Indeed, one can see that the *mdx* mice show a significant increase in absolute heart weight (Figure 6A). At the same time, the relative heart weight of these animals (heart weight/body weight) does not change (Figure 6B), which is probably due to an increase in the body weight of dystrophin-deficient mice based on skeletal muscle hypertrophy (Figure 6C). Alisporivir administration leads to a normalization of the absolute heart weight, as well as a significant decrease in the heart weight/body weight ratio in *mdx* mice (Figure 6B). In addition, one should note that alisporivir does not affect the heart weight of wild-type mice but causes a decrease in weight gain in the animals (Figure 6C).

**Figure 7:**
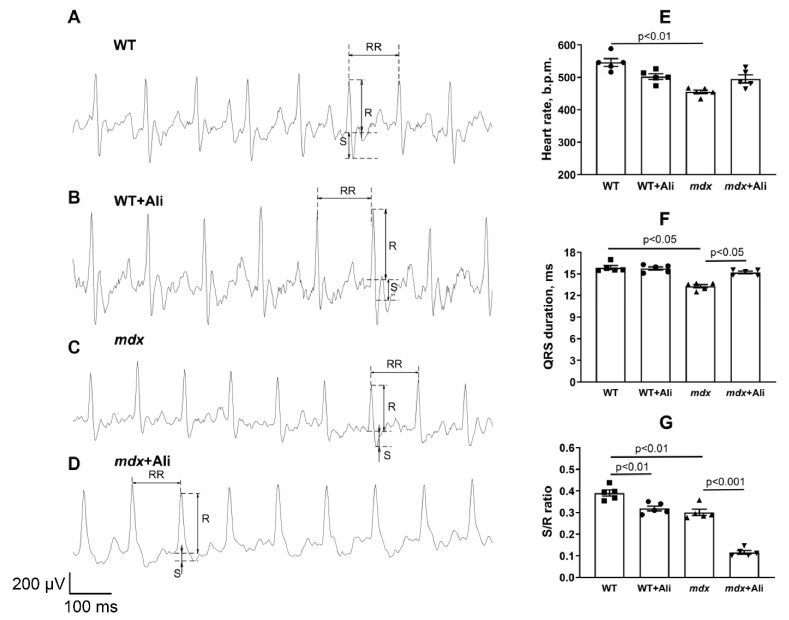
Representative ECG tracings from WT (**A**), WT + Ali (**B**), *mdx* (**C**), and *mdx* + Ali (**D**) groups of mice. Graphical representation of ECG profiles: heart rate (**E**), QRS duration (**F**), and S/R ratio (**G**). The data are presented as means ± SEM (*n* = 5).

Figure 7 shows representative ECG recordings of the studied groups of mice. We have noted the development of bradycardia—a decrease in heart rate (by 1.2 times)—in dystrophin-deficient animals (Figure 7E). In this case, treatment with alisporivir does not affect this parameter. It can be seen that *mdx* mice are characterized by a significantly shorter QRS duration, reflecting ventricular contraction (depolarization) of the heart’s electrical conduction system (Figure 7F). Moreover, we noted a decrease in the S/R wave ratio in the heart of *mdx* mice compared to wild-type mice, indicating a slower ventricular excitation (Figure 7G). Alisporivir administration results in a widening of the QRS duration in *mdx* mice. We also noted a significant decrease in the S/R wave ratio in the hearts of mice of both groups treated with alisporivir. This indicates a significant change in the process of transfer of excitation from the atria to the ventricles in mice of both groups receiving alisporivir.

## 4. Discussion

Improving mitochondrial function is one of the promising therapeutic strategies in DMD, which has been shown both in animal models and in biopsies from dystrophic patients [9,10,11,12,30]. In particular, one of the attractive approaches is the regulation of Ca^2+^ homeostasis, including the suppression of the MPT pore opening in the inner membrane of organelles. Currently, cyclophilin D is the only mitochondrial protein that, on the one hand, has been proven to be involved in initiating pore opening, and, on the other hand, specific agents blocking its activity have been selected that prevent MPTP initiation [31,32]. The first such compound was CsA exhibiting a desensitizing effect on MPT pore opening in vitro, as well as in some in vivo models [3], but it has no positive effect on the state of DMD patients [33], which seems to be associated with the immunosuppressive effect of this agent, as well as the ability to inhibit calcineurin signaling secondarily reducing myotube differentiation and muscle regeneration. Promising effects were shown for the non-immunosuppressive CsA analog alisporivir, which fails to inhibit calcineurin signaling and also partially rescues the dystrophic phenotype in *mdx* mice [9,10,11], a zebrafish model of DMD [12], and muscle biopsies from DMD patients [12]. Indeed, one can see that alisporivir administration leads to a decrease in the intensity of the inflammatory process in *mdx* mice, as evidenced by a decrease in serum AST level (Table 2).

It should be noted that DMD also shows the development of a cardiac phenotype. This is also typical for *mdx* mice, and although this model has been criticized for the late-onset cardiac phenotype (myocardial fibrosis is apparent from 6 months of age), it should be noted that, at 1 month of age, *mdx* mice exhibit early intolerance to dobutamine stress [34]. One should note that the effect of alisporivir-based therapy on cardiac function in DMD is currently unknown. This is especially important given the vital role of calcium ions in the activation of heart muscle contraction and the specificity of the functioning of the heart mitochondria in dystrophin-deficient mice. Indeed, normally these organelles are considered to play an auxiliary role in the regulation of calcium homeostasis in cardiomyocytes; the main role is assigned to the sarcoplasmic reticulum [35,36]. However, DMD conditions reveal a significant inhibition of the calcium-regulatory function of the sarcoplasmic reticulum [37], and in this case, the mitochondria exhibit hyperfunctionalization, which seems to compensate for the dysregulation of calcium homeostasis in cardiomyocytes, ensuring the organ’s adaptation to new conditions. This, in particular, manifests itself in an increase in the intensity of oxidative phosphorylation and the ability to transport and retain calcium ions in the matrix of organelles.

According to the results obtained in this work, the heart mitochondria of 12-week *mdx* mice show a tendency to disruption of the structural organization (but not the mitochondrial mass) (Figure 1 and Figure S1e) but still demonstrate an increase in the efficiency of oxidative ATP synthesis compared to WT animals (Table 3). The reason for this hyperfunctionalization of organelles may be an increase in the level of complex I of the respiratory chain, as well as ATP synthase, as evidenced by Western blot analysis of OXPHOS complexes (Figure 2). Indeed, the activity of complex I is known to change under DMD conditions, and, in particular, a decrease in its level in the skeletal muscle mitochondria was described, causing suppression of oxidative phosphorylation in skeletal muscles [38]. We observe the opposite picture in the case of heart mitochondria. One could speculate that this effect is primarily due to enrichment in complex I and ATP synthase due to the increase in the amount of Ca^2+^ in the mitochondrial matrix promoting the activation of mitochondrial dehydrogenases and the accumulation of NADH, the substrate of complex I. In this case, the 4-week alisporivir treatment shows a slight tendency to normalize the morphology and functional activity of cardiac mitochondria in *mdx* mice. However, it should be noted that these data will require confirmation in a larger cohort and other concentrations of alisporivir. At the same time, alisporivir practically does not affect the content of the respiratory chain complexes in the mitochondria of both groups of mice.

In addition, the heart mitochondria of 12-week *mdx* mice show an increase in calcium capacity (Figure 3A,B), which is consistent with previous findings [16] and suggests that mitochondria of *mdx* mice are more resistant to MPT pore induction. On the one hand, this may be associated with an increase in the efficiency of respiration of organelles, which is necessary for the transport of calcium ions into the matrix, or with a change in the level of proteins involved in the pore opening. However, as shown by us earlier [16] and confirmed in this work, the level of the regulatory protein of the MPT pore cyclophilin D and the possible channel-forming protein ANT2 does not change in the heart of *mdx* mice (Figure 3D,E). An exception is ANT1, but its level, on the contrary, increases in *mdx* animals (Figure 3C). It should be noted here that an increase in the expression of ANT1 can have a cardioprotective effect associated with an increase in the rate of adenine nucleotide transport [39]. This, in particular, may cause an increase in the functional activity of the heart mitochondria in dystrophin-deficient animals. In addition, an increase in resistance to MPT pore opening in the heart of *mdx* mice may be due to an increase in the microviscosity of mitochondrial membranes, which we revealed earlier [16]. Administration of alisporivir to *mdx* mice practically does not change the calcium capacity of cardiac mitochondria and does not affect the expression of genes encoding CypD, ANT1, and ANT2 proteins (Figure 3).

We previously showed that an increase in the respiration rate of heart mitochondria in *mdx* mice is accompanied by an increase in ROS generation by organelles [16], which may contribute to the development of oxidative stress, delayed organelle dysfunction, and cardiomyopathy. This is also observed in 12-week *mdx* animals, and we noted an increase in the level of lipid peroxidation products in the mitochondria of dystrophin-deficient animals (Figure 4). In this case, the use of alisporivir has no effect on the intensity of this process in both *mdx* mice and wild-type animals.

Modification of calcium homeostasis in mitochondria is also thought to be associated with altered organelle dynamics and mitochondrial biogenesis [40]. However, we did not notice a significant change in the expression of the *Drp1* and *Mfn2* genes regulating the intensity of mitochondrial fission and fusion (Figure 5A,B). At the same time, the heart of 12-week *mdx* mice shows signs of a decrease in the intensity of biogenesis; in particular, the expression of the transcription factor *Ppargc1a* is significantly reduced (Figure 5C). Reduced expression of *Ppargc1a* is also known to contribute to heart muscle dysfunction [41]. In this case, alisporivir administration leads to further suppression of organelle biogenesis in the heart of *mdx* mice, as evidenced by a decrease in *Ppargc1a* expression (Figure 5C) and a significant decrease in mtDNA level (Figure 1E) compared to vehicle-treated *mdx* mice. Moreover, we also noted a downward trend in *Ppargc1a* expression in wild-type mice, which is consistent with our recent data [42]. Thus, alisporivir suppresses mitochondrial biogenesis in the heart. It was previously shown that CsA is also able to suppress mitochondrial biogenesis [43], indicating not only their structural but also functional similarity.

The results obtained indicate that alisporivir, on the one hand, have no significant effect on the functional activity of heart mitochondria in *mdx* mice, but, on the other hand, suppresses organelle biogenesis, which is necessary for renewal of the mitochondrial population in the heart muscle and normal organ function. What are the consequences of chronic alisporivir administration for DMD correction in the case of cardiac phenotype? Our results suggest that at the organ level, its use may contribute to a reduction in heart weight increasing in DMD. Indeed, the weight of this organ is reduced in alisporivir-treated *mdx* animals (Figure 6A,B). Moreover, we noted a trend towards the recovery of heart rate in *mdx* animals treated with this drug (Figure 7E). All this indicates a positive trend. However, we found that the effects of alisporivir are accompanied by a significant modification of the transmission of excitation from the atria to the ventricles in *mdx* mice (Figure 7). Moreover, this effect of alisporivir is also observed in wild-type mice. The results obtained also indicate the complex effect of this agent on the heart muscle. It is interesting to note that chronic inhibition of cyclophilin D by CsA may be associated not only with inhibition of MPT pore opening but also accompanied by metabolic reprogramming and may have a negative effect on heart function [44]. The chronic effect of alisporivir may also be accompanied by similar rearrangements that affect the parameters of the work of this vital organ.

## 5. Conclusions

The nature of the development and rapid progression of skeletal muscle dysfunction is considered to require early initiation of therapy and, in particular, the use of a mitochondria-targeted approach based on alisporivir to support DMD patients may be justified already in the early stages of the disease. However, it is not known what effect the drug will have on a relatively stable cardiac phenotype in the early stages of the disease and what the prospects are for using this agent in the long term in DMD. This issue requires additional research.

## Figures and Tables

**Table 1:** List of gene-specific primers for RT-PCR analysis.

Gene	Forward (5′ → 3′)	Reverse (5′ → 3′)
*Ant1*	CTATGACACTGCCAAGGGGATG	TCAAACGGATAGGACACCAGC
*Ant2*	TCTGGACGCAAAGGAACTGA	GACCATGCGCCCTTGAAA
*Ppif*	GCAGATGTCGTGCCAAAGACTG	GCCATTGTGGTTGGTGAAGTCG
*Drp1*	TTACAGCACACAGGAATTGT	TTGTCACGGGCAACCTTTTA
*Mfn2*	CACGCTGATGCAGACGGAGAA	ATCCCAGCGGTTGTTCAGG
*Ppargc1a*	CTGCCATTGTTAAGACCGAG	GTGTGAGGAGGGTCATCGTT
*Rplp2*	CGGCTCAACAAGGTCATCAGTGA	AGCAGAAACAGCCACAGCCCCAC
*Nd4*	ATTATTATTACCCGATGAGGGAACC	ATTAAGATGAGGGCAATTAGCAGT
*Gapdh*	GTGAGGGAGATGCYCAGTGT	CTGGCATTGCTCTCAATGAC

**Table 2:** The level of creatine kinase, LDH and AST in the serum of experimental groups of mice.

Groups	Creatine Kinase	LDH	AST
	U/L		
WT (*n* = 10)	349.7 ± 74.4	349.5 ± 43.0	62.3 ± 9.9
WT + Ali (*n* = 9)	460.5 ± 79.8	260.0 ± 36.1	40.4 ± 7.6
*mdx* (*n* = 8)	3376.7 ± 670.6 *^#^	1355.4 ± 264.7 *^#^	414.0 ± 63.2 *^#^
*mdx* + Ali (*n* = 10)	2103.8 ± 316.7 *^#^	950.9 ± 106.1 *^#^	252.3 ± 23.5 *^#¥^

Data are presented as means ± SEM; *n*, the number of experimental animals. * *p* < 0.05 versus control group (WT); ^#^ *p* < 0.05 versus WT+Ali group; ^¥^ *p* < 0.05 versus *mdx* group.

**Table 3:** Effect of alisporivir on the parameters of respiration and oxidative phosphorylation of heart mitochondria of studied groups of mice.

Animal (*n* = 5)	State 2	State 3	State 4	State 3U_DNP_	RC	ADP/O
	nmol O_2_/min per 1 mg of Protein				Relative Units	
WT	11.4 ± 3.0	37.6 ± 2.9	14.6 ± 1.5	38.3 ± 3.3	2.6 ± 0.2	2.2 ± 0.1
WT + Ali	17.1 ± 1.6	52.5 ± 4.3	15.3 ± 1.0	50.1 ± 3.8	3.4 ± 0.2	2.5 ± 0.2
*mdx*	14.3 ± 0.9	60.5 ± 7.3 *	17.4 ± 2.0	63.7 ± 7.6 *	3.5 ± 0.2 *	2.5 ± 0.1
*mdx* + Ali	15.4 ± 1.2	53.7 ± 4.1	15.7 ± 1.3	53.5 ± 4.0	3.4 ± 0.2	2.3 ± 0.1

Medium composition: 120 mM KCl, 5 mM NaH_2_PO_4_, and 10 mM HEPES-KOH buffer (pH 7.4) Respiration of mitochondria was fueled by 2.5 mM glutamate + 2.5 mM malate. Respiration of mitochondria in state 3 was initiated by 200 μM ADP. Data are presented as means ± SEM; *n*, the number of experimental animals. * *p* < 0.05 versus control group (WT).

## Data Availability

The data presented in this study are available on request from the corresponding author.

## Supplementary Materials

The following are available online at https://www.mdpi.com/article/10.3390/biomedicines9091232/s1, Figure S1: Typical electron micrographs of left ventricular mitochondria in the experimental groups: WT (A,B), WT + Ali (C,D), *mdx* (E,F) and *mdx* + Ali (G,H). Samples from two hearts were analyzed in each experimental group. The bar is equal to 1 μm. Red arrows indicate individual abnormal mitochondria.
