# Supplementary material for: Effect of the Non-Immunosuppressive MPT Pore Inhibitor Alisporivir on the Functioning of Heart Mitochondria in Dystrophin-Deficient mdx Mice
Source: Biomedicines. 2021 Sep 16;9(9):1232. doi: 10.3390/biomedicines9091232 (PMC8466941; doi:10.3390/biomedicines9091232)
Supplement: Supplementary file 1 [file biomedicines-09-01232-s001.zip › biomedicines-1354940-supplementary.pdf]

biomedicines

MDPI

Supplementary Material: Effect of the Non-immunosuppressive MPT pore Inhibitor Alisporivir on the Functioning of Heart Mitochondria in Dystrophin-deficient *mdx* Mice

Mikhail V. Dubinin [1,*], Vlada S. Starinets [1,2], Eugeny Yu. Talanov [2], Irina B. Mikheeva [2], Natalia V. Belosludtseva [2], Dmitriy A. Serov [3], Kirill S. Tenkov [1], Evgeniya V. Belosludtseva[1], and Konstantin N. Belosludtsev [1,2]

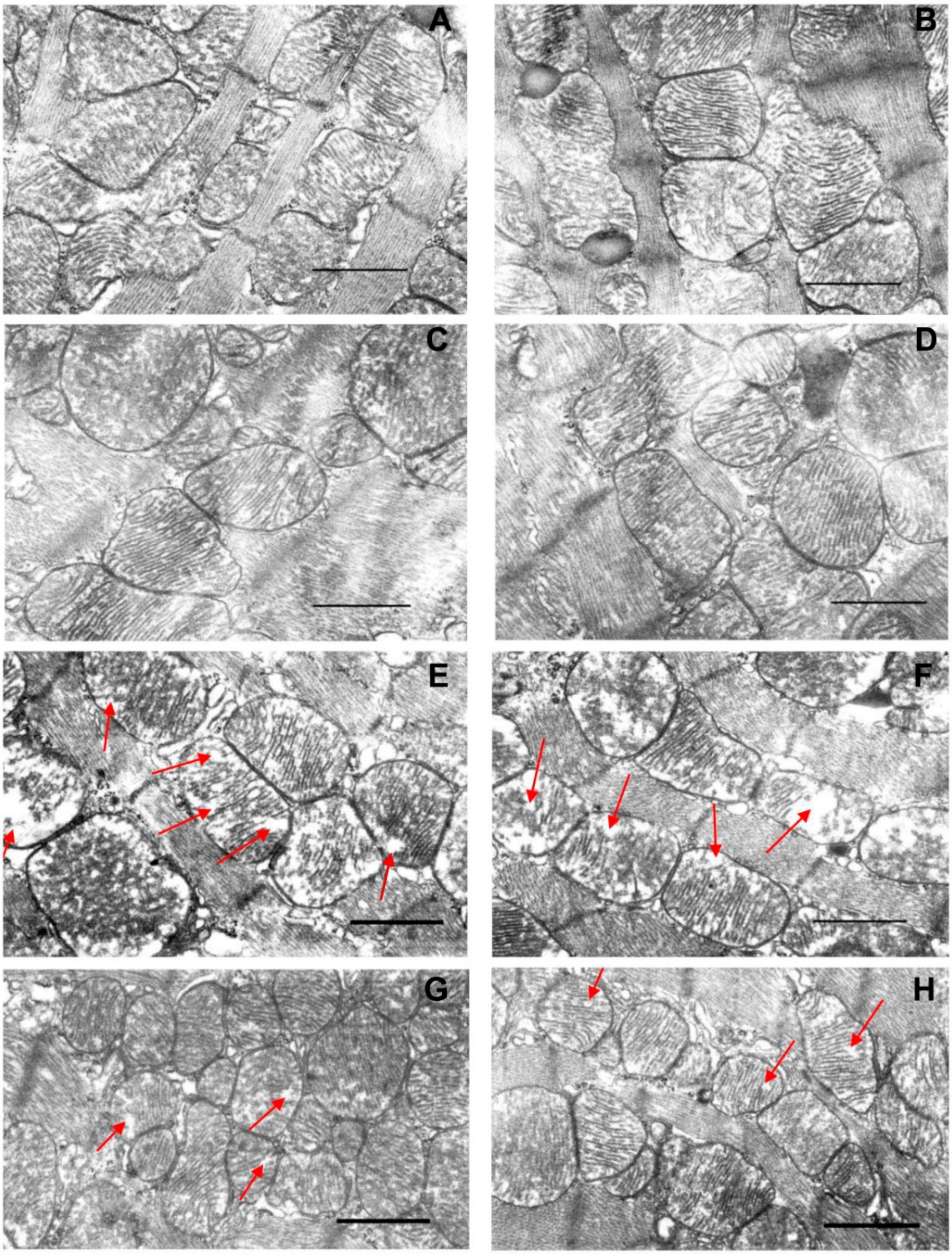

Figure S1. Typical electron micrographs of left ventricular mitochondria in the experimental groups: WT (A,B), WT+Ali (C,D), *mdx* (E,F) and *mdx*+Ali (G,H). Samples from two hearts were analyzed in each experimental group. The bar is equal to 1 μm. Red arrows indicate individual abnormal mitochondria.
